# Factors that influence parental satisfaction with SDF treatment in healthy children and those with special healthcare needs

**DOI:** 10.3389/fdmed.2023.1286162

**Published:** 2023-11-14

**Authors:** Y. O. Crystal, J. H. Jang, M. N. Janal, V. H. Raveis

**Affiliations:** ^1^ Department of Pediatric Dentistry, New York University College of Dentistry, New York, NY, United States; ^2^ Department of Epidemiology and Health Promotion, New York University College of Dentistry, New York, NY, United States; ^3^ Psychosocial Research Unit on Health, Aging and the Community (PRUHAC), Department of Cariology and Comprehensive Care, New York University College of Dentistry, New York, NY, United States

**Keywords:** silver diamine fluoride (SDF), dental caries, parental satisfaction, special healthcare needs, caries arrest, caries management, parental acceptance

## Abstract

**Purpose:**

Silver diamine fluoride (SDF) is used as a caries management agent for the arrest of dentinal caries lesions. The purpose of this study was to evaluate the satisfaction with SDF treatment provided at a university pediatric dentistry clinic and to identify factors that may contribute to parental dissatisfaction.

**Methods:**

We obtained retrospective data of children who received SDF treatment at our clinic from 1 February 2019 to 28 February 2021. Parents were contacted by phone to participate in a survey that evaluated their satisfaction with the treatment. Satisfaction was evaluated as a function of medical status, ease of treatment, outcome of SDF treatment, esthetics, and understanding of treatment goals and side effects using contingency tables and chi-square statistics.

**Results:**

From 209 children who received SDF treatment, we were able to contact 91 parents by telephone, and 79 agreed to participate. Special healthcare needs (SHCN) patients were overrepresented in our sample, comprising 22.3% of all treated and 36.7% of participants. More than 90% were satisfied with the treatment, would do it again and would recommend it to others. Among the 49 children who complained of pain, SDF treatment resolved 82% of these complaints. In the subsample with follow-up in our clinic, approximately half of the treated teeth later received restorative treatment or were extracted, and the other half presented without further treatment. Some children received further treatment elsewhere. Parental dissatisfaction was related to staining of the anterior teeth (*p* = 0.04), the need for further treatment (*p* = 0.02) and a lesser understanding of side effects (*p *= 0.002).

**Conclusion:**

Most parents were satisfied with SDF therapy as a dental treatment choice due to its easy application and desensitizing effects. Our findings indicate that parental understanding of the interim nature of the treatment and staining of the lesions is important to achieve parental satisfaction.

## 1. Introduction

Silver diamine fluoride (SDF) has been adopted and used in the dental community to arrest dentinal caries on primary teeth, apart from its FDA-approved purpose of treating dental hypersensitivity in adults. This off-label use has been supported in the American Academy of Pediatric Dentistry's (AAPD) 2017 Guideline for the use of SDF in children and adolescents including those with special needs (1). The guideline recommends 38% SDF for the arrest of dentinal caries in primary teeth as part of a comprehensive caries management plan. SDF has been included in the list of WHO essential medicines (2), and in the United States, its status as breakthrough therapy has initiated steps towards a change in labeling as a medicament for caries arrest (3).

Systematic reviews conclude that SDF is safe, effective, efficient, timely, patient centered, and equitable (4). The application of SDF is minimally invasive as it does not require caries removal, and therefore no local anesthetic. It provides dentists with an alternative to deliver safe and effective dental treatment to young children with limited cooperation who would otherwise require advanced forms of behavior management to provide traditional restorative treatment. The main downside of this easy-to-provide treatment is that although it arrests dentin and enamel lesions, the arrest is characterized by a black staining that can be visible depending on the location of the cavity. This esthetic problem can be a deterrent for parents' acceptance of this treatment option.

Many studies have evaluated parental perceptions and acceptance of SDF in reference to the staining. Our previous work, performed in the New York City metropolitan area, explored parental perceptions and acceptance of SDF when looking at parameters of esthetics and child's behavior using a hypothetical situation where parents were shown before and after pictures of SDF-treated teeth (5). Depending on the child's level of cooperation and the location of the treatment (anterior vs. posterior), most parents leaned towards accepting SDF therapy as their child would require more advanced pharmacological management for traditional restorative treatment, with higher acceptance for the posterior region where esthetics were not compromised.

Our subsequent work focused on parental concerns about SDF treatment (6). Six themes emerged from the comments parents brought up when they viewed pictures of treated teeth, including, “esthetic concerns, psychosocial concerns, SDF treatment process, risks and side effects, situational benefits, and dental treatment process”. The most prevalent concerns that arose were the esthetics and the psychosocial impacts it could have on their child. Their responses revealed the sources of parental hesitation in accepting this treatment which enables dental practitioners to address such concerns during the consent process.

Several other quality improvement projects (7), clinical trials (8, 9), and systematic reviews (10) that evaluate parental satisfaction with SDF therapy report that most parents were satisfied with the treatment despite the staining due to the ease of performing this treatment. Some of these published studies were done in places where cultural acceptance of esthetics could be different than in the United States. Also, studies done through clinical trials, where the treatment was provided in a school setting may not be generalizable to a private clinical scenario, where the level of parental involvement in the decision-making and their expectations on the outcomes are often very high.

Even with all published studies, there remains a need to explore parental satisfaction after parents have had the opportunity to see the short- and long-term effects of the SDF treatment. With reported arrest rates that range from 90% (11) to <40% (12), it is important to understand if parental satisfaction is related not only to the ease of treatment and esthetics but also to the success of the treatment and the eventual outcome of the treated teeth. It is also important to explore if there are other factors that influence parental satisfaction with SDF treatment. This knowledge would allow clinicians to improve their case selection and case presentation and to better advocate SDF for those candidates who can best benefit from this treatment.

The aim of this study was to evaluate parental satisfaction with SDF treatment, after it was provided to children in our clinic, and to identify the factors that may have influenced parental dissatisfaction.

## 2. Methods

This investigation was reviewed and approved by the Institutional Review Board (NYUIRB Study #S19-01393). Participants were recruited from the New York University College of Dentistry (NYUCD) Pediatric Dental clinic database where we obtained a list of records of children who received SDF between 1 February 2019 and 28 February 2021. The inclusion criterion was any child who received SDF treatment in our clinic, identified by CDT code, during those dates. No exclusions were applied.

We designed a data collection instrument to obtain information from the children's parents on the ease of treatment, the parent's report on their child's experience with SDF therapy, parent's recollection of the outcome of the tooth/teeth treated, their understanding of the benefits/side effects of SDF therapy, and their level of satisfaction with the treatment. We also abstracted from the children's chart variables that included gender, age, phone number, general health status, dental insurance, tooth/teeth treated with SDF, the outcome of the tooth if available and their last visit at our clinic. The questionnaire, in English and Spanish, was tested for clarity, precision and timing, by itself, and then with the recruitment statement and consent information required by the IRB. Testing was done with three volunteer parents, three dental assistants (who are also mothers) and two dentist faculty members. Changes were done until consensus from all authors was reached. The questionnaire is included in Table 1.

**Table 1:** SDF study questionnaire.

1. How did the visit go compared to other medical or dental visits your child has had?					
better		similar	worse		
2. Do you think the treatment was easy to do?					
yes			no		
3. Did your child complain of tooth pain after applying silver fluoride treatment?					
yes			no		
4. Did the treatment solve your child's tooth pain?					
yes		No	My child did not have previous pain		
5. What happened to the tooth/teeth treated with silver fluoride?					
*Tooth 1*	same	filling	cap/crown	pulled out	fell out
*Tooth 2*	same	filling	cap/crown	pulled out	fell out
*Tooth 3*	same	filling	cap/crown	pulled out	fell out
6. When they explained the silver fluoride treatment to you at our clinic before doing it, did you fully understand the benefits?					
yes			no		
7. And, did you fully understand the side effects?					
yes			no		
8. Are you bothered by the tooth staining?					
yes			no		
9. Do you think the staining bothers your child?					
yes		no	if yes, please elaborate……..		
10. Did this treatment meet your expectations?					
yes (please explain how……………..)			no (please explain how……………….)		
11. If your child had another cavity, would you choose this treatment again?					
yes, definitely		probably yes	probably no		no
12. Would you recommend this treatment to other parents?					
yes		probably	uncertain		no
13. Where do you regularly take your child to the dentist?					
NYU		hospital clinic	local private dentist		no regular dentist
14. What is the reason for any of the choices above?					
too far		I don’t have insurance	I have not had a chance to get an appointment		other
15. What is your age?					
16. What is the highest grade you completed?					
17. Do you have any other comments about the advantages or disadvantages of the treatment?					
Thank you for your time, 18. Do you have any final comments or anything you want to add?					

Parents were contacted by phone by a pediatric dental resident who speaks fluent English and a fourth year dental student who speaks fluent Spanish. In two cases where parents spoke a language other than English and Spanish (Arabic and Chinese), a phone interpreter was used to translate. Parents were read a recruitment statement with a description of the study and its benefits and risks. They were then asked to verbally consent to participate in the study. Parents who agreed to participate completed the questionnaire during the telephone interview. At the end of the questionnaire, parents were given the opportunity to comment or expand on their views of the treatment or the study. Numerous parents provided insightful comments after completing the telephone questionnaire. These comments were analyzed qualitatively and will be reported in a separate publication.

Responses to the questionnaire were collected and stored in REDCap (REDCap NYU Langone Health) and exported to IBM SPSS (v 28, IBM Corp, Armonk, NY). Analysis used the Crosstabs procedure to evaluate contingency tables and the chi-square statistic to determine the probability of chance associations. “Significance” indicates *p* < .05.

## 3. Results

From the 209 records that met the criteria, 91 parents were successfully contacted by telephone (44%), and 79 (87%) of those reached consented to participate in this research study. The response rate was 38% of all treated and 87% of those successfully contacted. The reasons that parents did not participate in the study were “no answer” or “phone number was invalid” (*n* = 118), or “reached, but declined to participate” (*n* = 12).

Demographic information on all children treated and children of parents who participated in the study is presented in Table 2.

**Table 2:** Demographics.

		All treated *n *= 209	Participants *n* = 79
Age (years)	1–2	39 (19%)	14 (18%)
	3–4	92 (44%)	33 (42%)
	5–6	51 (24%)	21 (26%)
	7>	27 (13%)	11 (14%)
Medical History	Special Healthcare Needs	48 (22.3%)	29 (36.7%)
Gender	Male	125 (59.8%)	47 (59.5%)
	Female	84 (40.2%)	32 (40.5%)

The results show a similar distribution of age and gender in the treated and the recruited samples, indicating that our sample population was representative of the total population of children treated. On the other hand, the special healthcare needs children (SHCN) were overrepresented in the recruited sample. The ethnicity of participants could not be reported because the disclosure of ethnicity in our records is voluntary. Most of the records obtained did not have this information. Of the parents contacted, 38 parents chose to answer the questionnaire in Spanish, 39 answered it in English, one participant requested an Arabic translator, and another participant requested a Chinese translator. This approximate ethnic breakdown is representative of the population seen at our clinic.

In terms of satisfaction with the treatment their child received, with the exception of one parent who reported being “very dissatisfied”, parents reported that they were either “satisfied” (*n* = 60) or “somewhat satisfied” (*n* = 18) with the treatment. Most parents (88.6%) would “probably” or “certainly” recommend this treatment to others, and most (92.1%) would “probably” or “definitely” choose this treatment again. Also, most (81.1%) rated the encounter when SDF was applied as “better than other medical or dental visits” and as “easy to do” (90%). Forty parents reported that SDF application successfully resolved their child's pain. Among the 49 children who complained of pain, SDF treatment resolved 82% of these complaints. It can be concluded that parents were generally satisfied with their child's treatment with SDF. These results are summarized in Table 3.

**Table 3:** Parental satisfaction.

Overall parental satisfaction	
Satisfied	60 (76%)
Somewhat satisfied	18 (23%)
Very dissatisfied	1 (1%)
Would recommend this treatment to others	70 (89%)
Would choose this treatment again	72 (92%)
Was better than other medical or dental visits	81%
Pre-operative pain	
Patients with pre-operative caries pain	49
SDF resolved their pain due to caries	40 (82%)

In terms of esthetics, 10 (13%) parents reported that they were bothered by the staining, and 5 (6%) reported that their child was bothered by the staining. Nine of the 10 children whose parents were bothered by the staining received treatment of anterior or anterior and posterior teeth (*p* = 0.02). All 5 children who were bothered by the staining received treatment of either anterior or anterior and posterior teeth (*p* = 0.04). Interestingly, parents who were bothered by the staining did not report lower levels of satisfaction than other parents. Thus, while few were bothered by the SDF staining, those who were had SDF placed in anterior teeth.

Eight parents (10%) reported that they did not fully understand the benefits of SDF therapy and 17 (22%) reported they did not fully understand the side effects of this therapy. Lesser understanding of the benefits was associated with a reduced probability of recommending the treatment to others (22% vs. 77.5%, *p* = 0.02). Lesser understanding of the side effects was also associated with lower reports of meeting expectations (70.6% vs. 96.8%, *p* = 0.004), choosing to receive another SDF treatment (35.3% vs. 74.2%, *p* < 0.001), and satisfaction with the treatment (41.2% vs. 85.5%, *p* = 0.002). Those open to treating future caries with SDF tended to report that the visit went “better than usual” compared to those reporting “similar” or “worse” visits (96.3% vs. 76.9% and 50%, *p* < 0.001). They also thought that the treatment was “easy to do” (92.9% vs. 75.0%, *p* = 0.02). These findings indicate that not fully understanding the side effects and, to a lesser extent, not fully understanding the benefits of SDF treatment negatively impacted satisfaction with the treatment.

While 273 teeth were treated in 79 children, follow-up clinic data were available on only 153 teeth in 47 children (60% of the sample). The clinic records indicated that, in this subsample, 75 teeth (49%) received further treatment: 21 restorations, 23 stainless steel crowns (SSCs), and 31 extractions. Of the remaining 78 teeth (in 32 children in the sample), 10 were exfoliated, and 68 (44.4%) presented with an SDF-treated tooth present. The need for further treatment (from the parent's recollection) was associated with reduced reports of meeting parental expectations (40% vs. 100%, *p* = 0.02), but no other indicators of satisfaction. Overall, in this subsample treated in our clinic, approximately half of the treated teeth later received restorative treatment or were extracted, and the other half presented without further treatment. Parental recollections may have included teeth that were treated in other clinics or in the OR that are not tallied in our clinic records. Regardless of where the teeth were treated, the need for further treatment went against parental expectations. Table 4 summarizes the factors that negatively affected satisfaction.

**Table 4:** Factors that negatively affected satisfaction.

Esthetics	
Parents were bothered by the staining *9 of these had anterior teeth treated*	10 (13%)
Children were bothered by the staining *5 of these had anterior teeth treated*	5 (6%)
Understanding of SDF treatment	
Parents did not understand the benefits	8 (10%)
Parents did not understand the side effects	17 (22%)
Outcome of the treatment	
Children who received further treatment in our clinic^a^	47 (60%)
Teeth that received further treatment in our clinic^a^	75 (49%)
Restorations	21
SSCs	23
Extracted	31
Teeth that presented without further treatment	78

^a^ Some children had treatment provided either at a hospital's operating room or in other clinics and their details are not included in these figures but could be part of the parents' recollection.

## 4. Discussion

Results from this study documented high levels of satisfaction among parents whose children were treated with SDF. SDF treatment resolved most of the caries-related pain problems, and a majority of parents reported that they would choose this treatment again and recommend it to others. Dissatisfaction was related primarily to a limited understanding about the need for further treatment and SDF therapy's side effects. There was little dissatisfaction related to tooth staining, but when it was expressed, it was related to treatment of the anterior teeth. The fact that parental dissatisfaction was related to both the need for further treatment and a lesser understanding of side effects points out the importance of emphasizing these issues during case presentation.

This study collected data from the records of children who received SDF at our clinic during the years 2019–2020, a time interval that was chosen to enable a follow-up of the final outcome of the tooth/teeth treated. We found that in some cases, it was difficult for parents to recall what procedures were done and/or the experience. However, once they were asked specifically about the effects of SDF, they easily remembered the treatment, as SDF leaves a marked black discoloration. This indicates that the majority of parents had a good understanding of the benefits and side effects of SDF therapy. Most of them were satisfied with SDF because they perceived it was the most appropriate option for their child, and in retrospect, they were not bothered by the staining. Our results support that satisfaction with SDF is higher when the discoloration is not in a very visible area. This is consistent with our previous studies (5, 6), as well as several others (10, 13). This is important for practitioners to keep in mind, as a recent scoping review concluded that, whereas parents were satisfied with and found SDF acceptable, professionals did not (14). Specifically, this review found that dental practitioners tended to shy away from offering this treatment choice owing to their automatic assumption that parents will reject SDF due to the black staining.

The response rate to our survey was 38% of all children treated and 87% of those who we were able to contact. The majority of parents of the children treated in our clinic who could not be contacted by phone had a phone number that was currently invalid or did not answer the call. Like many other university health centers in major cities, ours acts as a safety net clinic and many children come with referrals for a specific problem and then return to their referring doctor when their main complaint is resolved. Many of the families who come to our clinic do not have stable long-term phone contact and do not bring their children regularly for preventive visits. However, from those who we were able to contact, the majority consented to participate in the study.

An interesting finding about the demographics of our study is that a significant number of children treated and children whose parents participated in the study were children with SHCN (22.3% of all treated and 36.7% of participants). It is apparent that these children attend our clinic regularly; thus, their parents consider us their dental home. This is important as it is likely that this population has difficulty finding adequate providers elsewhere and is willing to travel for their child to receive sustained periodic care. It is also likely that we saw a high number of this population in our treated group and in our respondents, because minimally invasive procedures like SDF are a valuable tool for treating children with limited levels of cooperation (13). The value of this treatment is reflected in the fact that 81% of parents responded that their children had a better visit during the application of SDF when compared to other medical or dental visits, and 90% of participants reported the treatment was easy to do. These responses also capture the perception of parents of healthy children who are unable to cooperate in the delivery of traditional treatment because they are very young, pre-cooperative, or have fears.

In our clinic, many of the children receive SDF as an interim treatment until they receive definitive treatment under general anesthesia, as the waiting times for that clinic service can range from 3 to 5 months. We also recommend SDF for children who will receive traditional treatment, in instances where completion of that treatment will take multiple visits that may span several months, depending on the behavior and tolerance of the child and the compliance of the family for the visits. This is in line with what is taught at most pediatric dentistry programs in the United States (15). SDF can also be placed on teeth that are hyper-sensitive to de-sensitize the tooth prior to performing a definitive restorative treatment (16).

Although SDF is a great tool to treat children who have barriers to accepting more complicated treatments, it needs to be noted that it certainly has limitations. One of them is that when applied to teeth that have pulpal involvement, they can become necrotic and develop infections that need to be treated with extraction (17). In children who have a problem tolerating treatment, it is not always possible to obtain x-rays. Consequently, the diagnosis for a specific tooth can be questionable. However, SDF can still be the best and most appropriate option for these children. In these instances, the cases must be monitored closely, and parents must be advised that the final outcome may still be extraction of the tooth.

Another restriction of SDF is that its efficacy ranges from 40% to 90% based on the location of the cavity and the presence of plaque (11, 12, 18). The ability to keep the lesion free of plaque seems to be an important factor to ensure that lesions become and remain arrested. In children that cannot achieve this, or on lesions where this is not easily attainable, SDF can still be used as a first line of treatment, as it is an effective desensitizer, and it may allow the child or the parent to implement better hygiene measures in an area that was previously sensitive (16, 19). However, parents will need to understand that SDF will be followed by treatment that can keep the lesion free of plaque in order to stop the advance of tissue destruction by caries (20, 21). Subsequent treatment that will achieve this goal can be minimally invasive like glass-ionomer restorations, Hall-style SSCs, or traditional restorative treatment. Our results indicate that as long as parents understand the limitations and the possible need for additional treatment, they will find SDF a valuable therapy for the treatment of their children's oral health needs.

It is important to recognize that the high levels of satisfaction found in our study may be due to the fact that our sampled population had already previously consented to receive SDF treatment after a careful presentation that included before and after pictures of treated teeth, as well as alternative options for treatment. In our clinic, not all parents who were presented with the option of SDF treatment chose to accept it for their children, ultimately choosing other options.

Another limitation of our study is that we were unable to reach every parent of the children that we treated with SDF therapy. However, the careful analysis of the information we obtained from those who we were able to reach can be generalized to offer valuable information that will allow clinicians to improve patient selection and case presentation. Future investigations should ask parents more detailed questions about their expectations regarding the need for additional treatment following SDF to provide greater insights on their understanding of the short and long-term outcomes of SDF therapy.

## 5. Conclusions and relevance

(1) Satisfaction with SDF treatment was high due to its easy application and desensitizing effects.
(2) Treatment dissatisfaction was mainly related to the child's need for further treatment, a lesser understanding of side effects, and treatment of anterior teeth.
(3) SDF was frequently used in our clinic as an interim medicament for caries arrest. Most of these children subsequently received further treatment on their treated teeth. Case presentation should include not only the side effects (staining) and limitations of SDF but also clearly state the goals when using this therapy as interim treatment.

## Data Availability

The raw data supporting the conclusions of this article will be made available by the authors, without undue reservation.

## References

[1] Crystal YO Marghalani AA Ureles SD Wright JT Sulyanto R Divaris K Use of silver diamine fluoride for dental caries management in children and adolescents, including those with special health care needs. Pediatr Dent. (2017) 39(5):135–45. PMID: 29070149

[2] Organization WH. World Health Organization model list of essential medicines – 22nd List, 2021. Geneva: World Health Organization. (2021).

[3] ClinicalTrials.gov. Effectiveness of Silver Diamine Fluoride (SDF) in Arresting Cavitated Caries Lesions. NCT03649659. Available at: https://clinicaltrialsgov/ct2/show/NCT036496592018

[4] Crystal YO Niederman R. Evidence-based dentistry update on silver diamine fluoride. Dent Clin North Am. (2019) 63(1):45–68. 10.1016/j.cden.2018.08.011 30447792 PMC6500430

[5] Crystal YO Janal MN Hamilton DS Niederman R. Parental perceptions and acceptance of silver diamine fluoride staining. J Am Dent Assoc. (2017) 148(7):510–8.e4. 10.1016/j.adaj.2017.03.013 28457477 PMC6771934

[6] Crystal YO Kreider B Raveis VH. Parental expressed concerns about silver diamine fluoride (SDF) treatment. J Clin Pediatr Dent. (2019) 43(3):155–60. 10.17796/1053-4625-43.3.2 30964719

[7] Huebner CE Milgrom P Cunha-Cruz J Scott J Spiekerman C Ludwig S Parents’ satisfaction with silver diamine fluoride treatment of carious lesions in children. J Dent Child. (2020) 87(1):4–11. PIMD: 32151304

[8] Clemens J Gold J Chaffin J. Effect and acceptance of silver diamine fluoride treatment on dental caries in primary teeth. J Public Health Dent. (2018) 78(1):63–8. 10.1111/jphd.12241 28749529

[9] Zheng FM Yan IG Duangthip D Lo ECM Gao SS Chu CH. Caries prevention using silver diamine fluoride: a 12-month clinical trial. Int Dent J. (2023) 73(5):667–73. 10.1016/j.identj.2022.12.005 36682908 PMC10509425

[10] Sabbagh H Othman M Khogeer L Al-Harbi H Al Harthi A Abdulgader Yaseen Abdulgader A. Parental acceptance of silver diamine fluoride application on primary dentition: a systematic review and meta-analysis. BMC Oral Health. (2020) 20(1):227. 10.1186/s12903-020-01195-3 32819333 PMC7439720

[11] Fung MHT Duangthip D Wong MCM Lo ECM Chu CH. Randomized clinical trial of 12% and 38% silver diamine fluoride treatment. J Dent Res. (2018) 97(2):171–8. 10.1177/0022034517728496 28846469 PMC6429575

[12] Mabangkhru S Duangthip D Chu CH Phonghanyudh A Jirarattanasopha V. A randomized clinical trial to arrest dentin caries in young children using silver diamine fluoride. J Dent. (2020) 99:103375. 10.1016/j.jdent.2020.103375 32428523

[13] Almarwan M Almawash A AlBrekan A Albluwi S. Parental acceptance for the use of silver diamine fluoride on their special health care-needs Child's primary and permanent teeth. Clin Cosmet Investig Dent. (2021) 13:195–200. 10.2147/CCIDE.S307190 34054310 PMC8153067

[14] Magno MB Silva LPD Ferreira DM Barja-Fidalgo F Fonseca-Gonçalves A. Aesthetic perception, acceptability and satisfaction in the treatment of caries lesions with silver diamine fluoride: a scoping review. Int J Paediatr Dent. (2019) 29(3):257–66. 10.1111/ipd.12465 30637833

[15] Crystal YO Janal MN Yim S Nelson T. Teaching and utilization of silver diamine fluoride and Hall-style crowns in US pediatric dentistry residency programs. J Am Dent Assoc. (2020) 151(10):755–63. 10.1016/j.adaj.2020.06.022 32979954 PMC7510543

[16] Ballikaya E Unverdi GE Cehreli ZC. Management of initial carious lesions of hypomineralized molars (MIH) with silver diamine fluoride or silver-modified atraumatic restorative treatment (SMART): 1-year results of a prospective, randomized clinical trial. Clin Oral Investig. (2022) 26(2):2197–205. 10.1007/s00784-021-04236-5 34743243 PMC8572062

[17] Zaeneldin A Yu OY Chu CH. Effect of silver diamine fluoride on vital dental pulp: a systematic review. J Dent. (2022) 119:104066. 10.1016/j.jdent.2022.104066 35139409

[18] Duangthip D Wong MCM Chu CH Lo ECM. Caries arrest by topical fluorides in preschool children: 30-month results. J Dent. (2018) 70:74–9. 10.1016/j.jdent.2017.12.013 29289726

[19] Cernigliaro D Kumar A Northridge ME Wu Y Troxel AB Cunha-Cruz J Caregiver satisfaction with interim silver diamine fluoride applications for their children with caries prior to operating room treatment or sedation. J Public Health Dent. (2019) 79(4):286–91. 10.1111/jphd.12338 31418870

[20] Jiang M Wong MCM Chu CH Dai L Lo ECM. Effects of restoring SDF-treated and untreated dentine caries lesions on parental satisfaction and oral health related quality of life of preschool children. J Dent. (2019) 88:103171. 10.1016/j.jdent.2019.07.009 31325466

[21] Chai HH Chen KJ Duangthip D Lo ECM Chu CH Gao SS. Parental perspectives on the use of silver diamine fluoride therapy to arrest early childhood caries in kindergarten outreach dental services: a qualitative study. J Dent. (2022) 125:104250. 10.1016/j.jdent.2022.104250 35944874

